# Supplementary material for: Modelling the impact of COVID-19-related programme interruptions on visceral leishmaniasis in India
Source: Trans R Soc Trop Med Hyg. 2021 Feb 13;115(3):229–35. doi: 10.1093/trstmh/trab012 (PMC7928630; doi:10.1093/trstmh/trab012)
Supplement: trab012_Supplemental_File [file trab012_supplemental_file.docx]

Supplementary Information

**Modelling the impact of COVID-19 related programme interruptions on visceral leishmaniasis in India**

Epke A Le Rutte^a,b,c,*^, Luc E Coffeng^a^, Johanna Muñoz^a^, Sake J de Vlas^a^

^a^ Department of Public Health, Erasmus MC, University Medical Center Rotterdam, 3000 CA Rotterdam, The Netherlands,

^b^ Department of Epidemiology and Public Health, Swiss Tropical and Public Health Institute, Basel, Switzerland,

^c^ University of Basel, Basel, Switzerland

*Corresponding author, E-mail: [epke.lerutte@swisstph.ch](mailto:epke.lerutte@swisstph.ch)

# 1. Table of Contents

[1. Table of Contents 1](#_Toc54036084)

[2. Model structure 2](#_Toc54036085)

[3. Model parameters 2](#_Toc54036086)

[4. PRIME-NTD Summary Table 2](#_Toc54036087)

[5. Additional figures 3](#_Toc54036088)

[5.1 Predicted VL incidence over time for counterfactual scenarios 3](#_Toc54036089)

[5.2 Predicted VL incidence over time for three outcome scenarios 4](#_Toc54036090)

[5.3 Differences in time to achieving VL elimination for 3 alternative durations of interruption 5](#_Toc54036091)

[5.4 Differences in cumulative VL incidence for 3 alternative durations of interruption 7](#_Toc54036092)

[5. References 10](#_Toc54036093)

# 2. Model structure


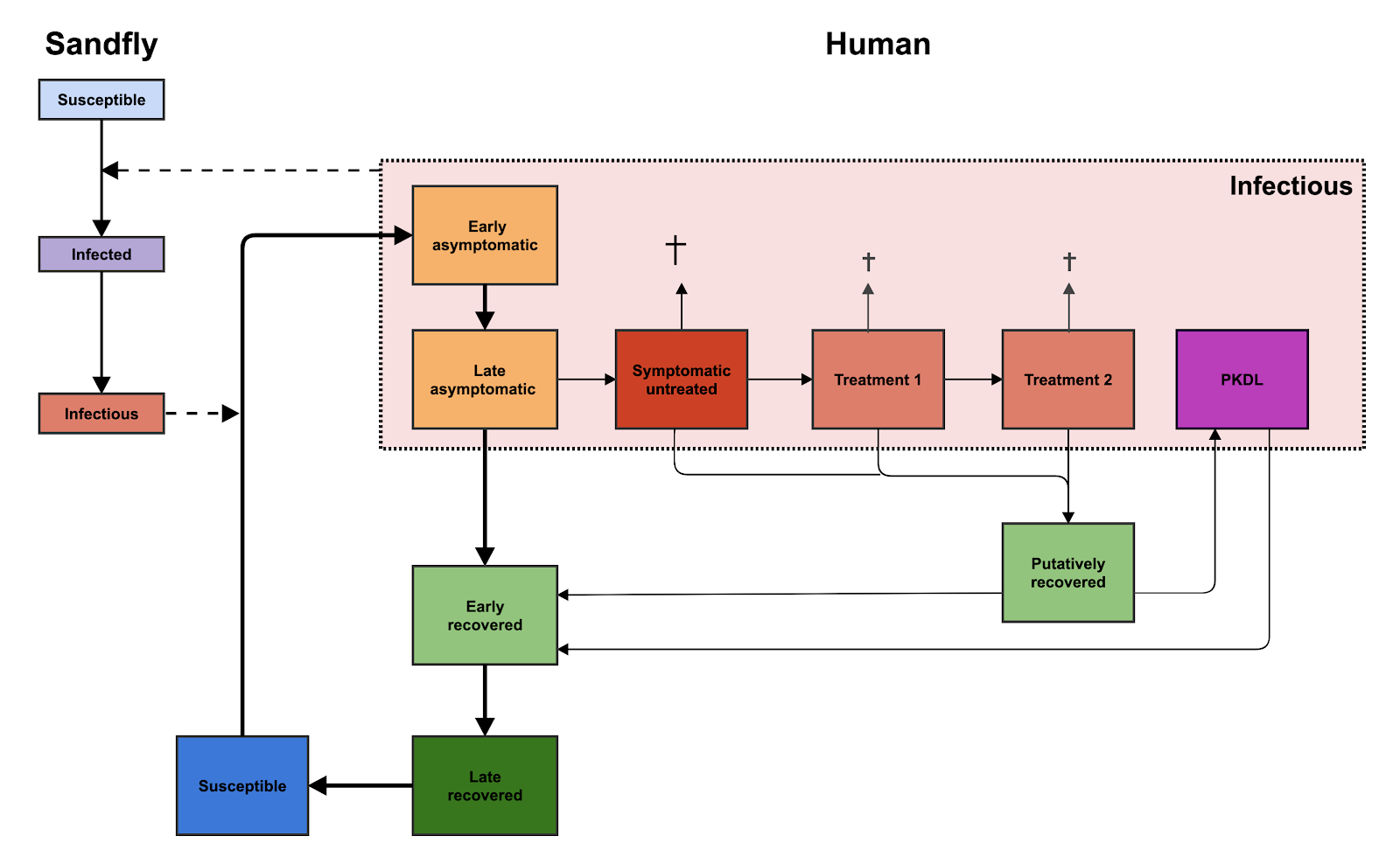


**Figure S1. Schematic presentation of the structure of model E1 and the related model E0.** For model E1, asymptomatic individuals (yellow compartments) are the main contributors to transmission. Model E0 has the same structure as model E1, but asymptomatic individuals do not contribute to transmission. Both models have different durations of infection stages from fitting to data, which are listed below in Chapter 3 Model parameters.

#

# 3. Model parameters

**Table S1. Parameter values**

**Table 1. Overview of parameter values and assumptions**

| **Parameters** | **Value^a^** | **Source** |
| --- | --- | --- |
| Human birth rate (per 1000 capita) | 21 (Indian crude birth rate in 2011) | ^1^ |
| Human mortality rate | Age-dependent (Indian mortality rates in 2011) | ^2^ |
| Average duration of early asymptomatic stage (days) | 202 | Fitted to data in ^3,4^ |
| Average duration of late asymptomatic stage (days) | 69 | Fitted to data in ^3,4^ |
| Average duration of symptomatic untreated stage (days) | 60 (pre-control), 45 (attack-phase), 30 (consolidation phase) | ^4–6^ |
| Average duration of symptomatic treatment 1 (days) | 1 | ^7^ (CHECK) |
| Average duration of symptomatic treatment 2 (days) | 28 | ^4,5,8^ |
| Average duration of putatively recovered stage (months) | 21 | ^9–11^ |
| Average duration of PKDL (years) | 5 | Expert opinion and ^10^ |
| Average duration of early recovered stage (days) | 236 | Fitted in ^3,4^ |
| Average duration of late recovered stage (years) | 2 | Assumption based on ^3^ |
| Relative infectiveness of early asymptomatic individuals | 0.0114 (Model E1) 0 (Model E0) | Fitted (E1) Pre-set (E0) |
| Relative infectiveness of late asymptomatic individuals | 0.0229(Model E1) 0 (Model E0) | Fitted (E1) Pre-set (E0) |
| Relative infectiveness of symptomatic untreated cases | 1 | Reference value |
| Relative infectiveness of patients under treatment 1 and 2 | 0.5 | Expert opinion and ^3^ |
| Relative infectiveness of PKDL cases | 0.9 | ^12,13^ |
| Fraction of late asymptomatic individuals that become symptomatic untreated | 1.4% | Fitted in ^3,4^ |
| Fraction of untreated symptomatic cases that spontaneously, putatively recover | 3% | ^14^ |
| Excess mortality rate among untreated symptomatic cases (per day) | 1/150 | Assumption |
| Excess mortality rate among treated symptomatic cases (per day) | 1/120 | Assumption ^7,8^ |
| Fraction of failed first-line treatments | 11% | Based on data presented in Supplementary File 2 of ^4^ |
| Fraction of putatively recovered cases that develop PKDL | 2.5 | ^4,15,16^ |
| Average life expectancy of the sandfly (days) | 14 | ^17,18^ |
| Average duration of incubation period in sandflies (days) | 5 | ^19^ |
| Sandfly biting rate (per day) | 0.25 | ^20,21^ |
| Transmission probability sandfly to human | 1.0^b^ | Reference value |

# ^a^ The parameter values listed here are the same for Models E0 and E1, unless stated otherwise.

^b^ The probability that a susceptible person becomes infected when bitten by an infectious sandfly is assumed to be 1; potential overestimation is compensated by the estimated sandfly density per human.

# 4. PRIME-NTD Summary Table

**Table S2. Policy-Relevant Items for Reporting Models in Epidemiology of Neglected Tropical Diseases (PRIME-NTD) Summary Table.** ^22^

| **Principle** | **What has been done to satisfy the principle?** | **Where in the manuscript is this described?** |
| --- | --- | --- |
| 1. Stakeholder engagement | Involved WHO HQ and PATH India | Introduction |
| 2. Complete model documentation | Described in detail in previous open access publications and on Github | Referred to previous papers in Methods, link to full open access of model code and documentation on Github in methods and here: [GITHUB LINK]. |
| 3. Complete description of data used | Described in detail in previous publications | Referred to particular datasets and previous papers in Methods^3,4^ |
| 4. Communicating uncertainty | Described in detail in previous publications and also highlighted in this paper | Methods^4^/discussion |
| 5. Testable model outcomes | Not yet, in the future the model predictions can be compared to KAMIS data. However, there is a discrepancy to be expected between real incidence and detected incidence. | Discussion |

# 5. Additional figures

## 5.1 Predicted VL incidence over time for counterfactual scenarios


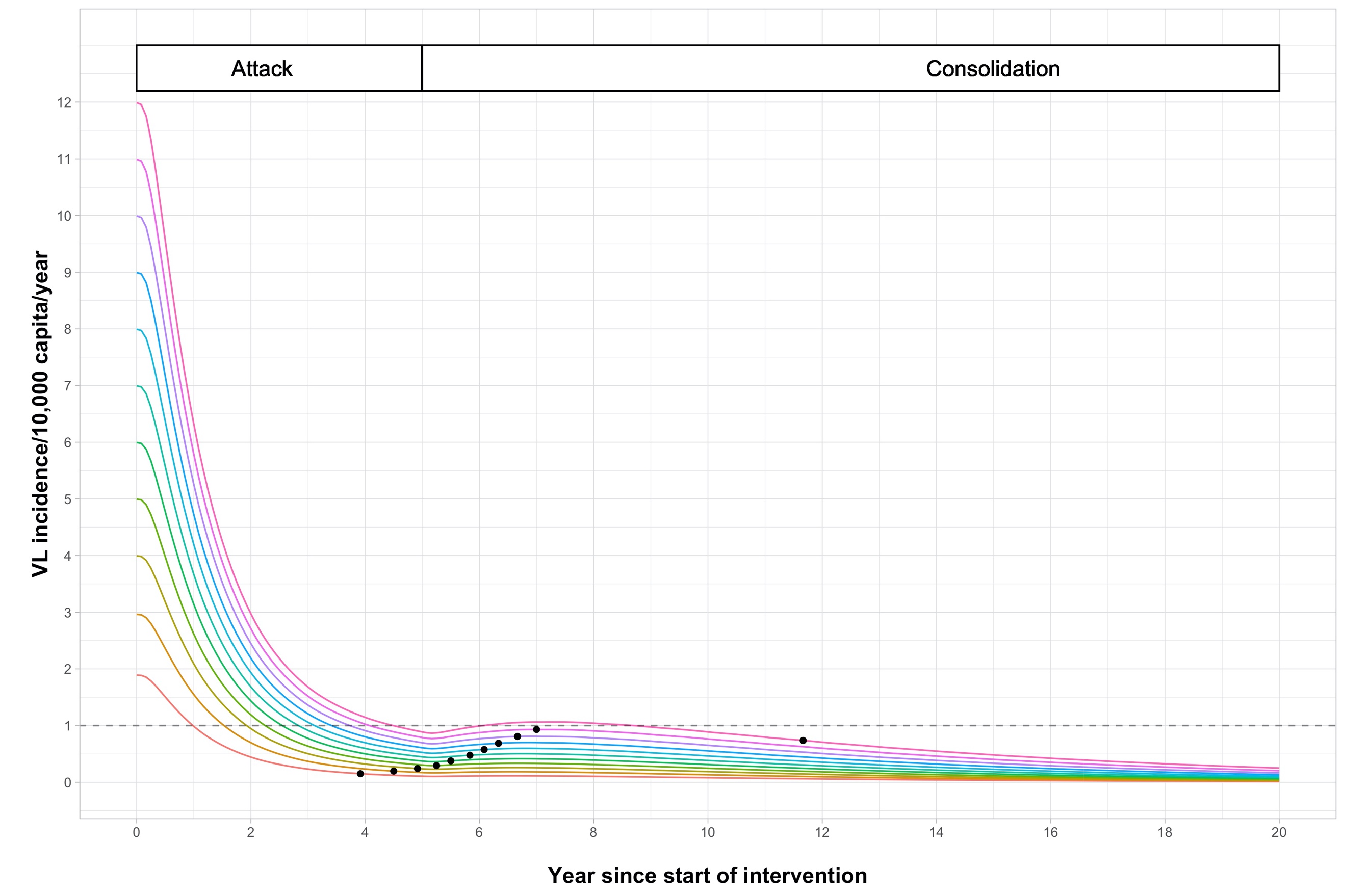


**Figure S2. Predicted visceral leishmaniasis incidence over time with expected times of achieving elimination (Model E1).** VL incidence is considered to be true incidence, so both detected and undetected cases. The coloured lines each represent a VL transmission setting with a different pre-control endemicity level. The white bars at the top stating ‘Attack phase’ and ‘Consolidation phase’ represent the course of the control strategy. The black dots represent the time of achieving elimination, which is defined as a VL incidence below 1 VL case per 10,000 people per year at sub-district level for 3 consecutive years.


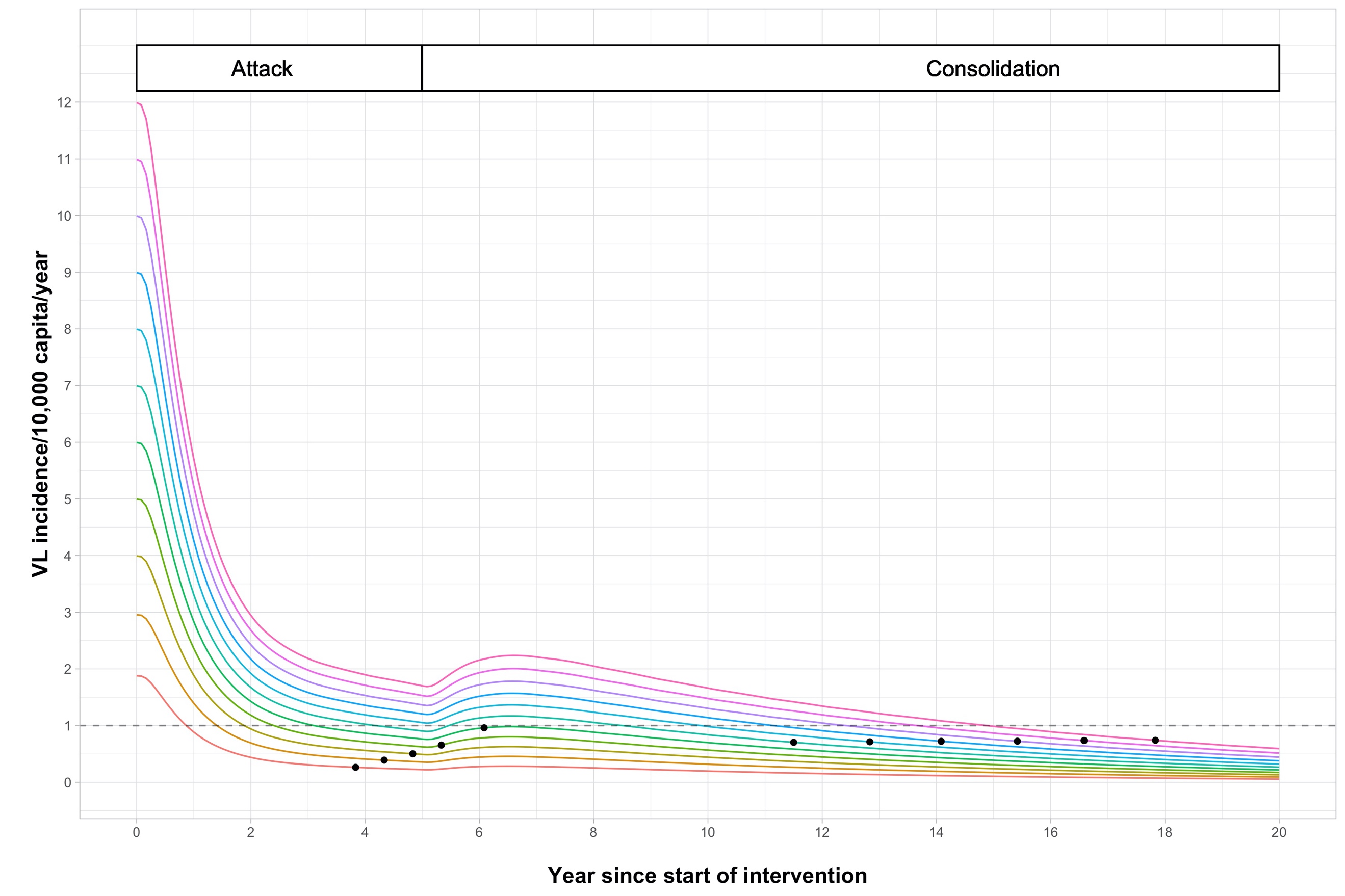


**Figure S3. Predicted visceral leishmaniasis incidence over time with expected times of achieving elimination (Model E0).** VL incidence is considered to be true incidence, so both detected and undetected cases. The coloured lines each represent a VL transmission setting with a different pre-control endemicity level. The white bars at the top stating ‘Attack phase’ and ‘Consolidation phase’ represent the course of the control strategy. The black dots represent the time of achieving elimination, which is defined as a VL incidence below 1 VL case per 10,000 people per year at sub-district level for 3 consecutive years.

5.2 Predicted VL incidence over time for three outcome scenarios


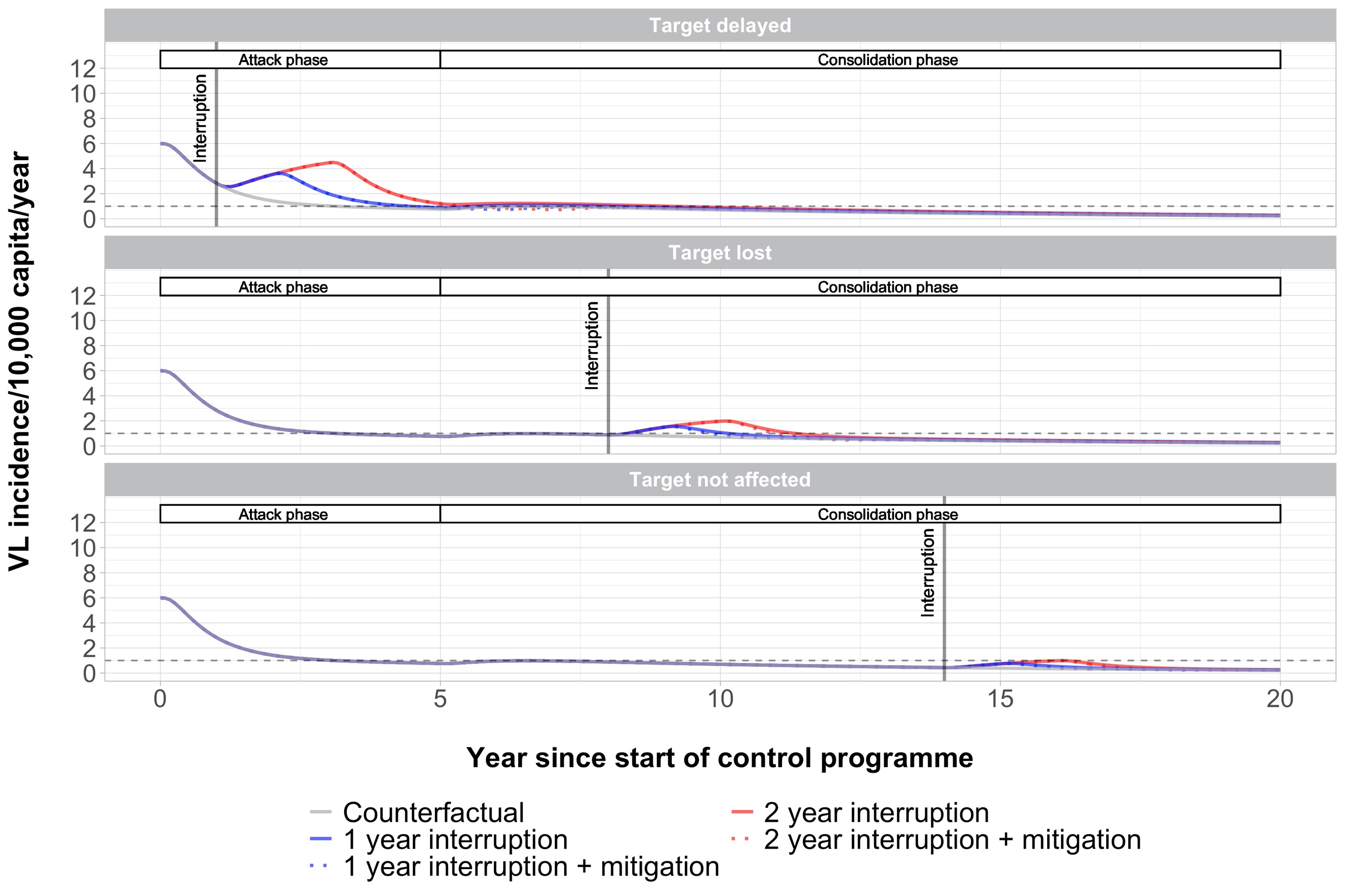


**Figure S4. Predicted visceral leishmaniasis incidence over time by Model E0.** Three interruption scenarios are presented for a setting with a pre-control endemicity of 6 VL cases/10,000/year. The white bars at the top stating ‘Attack phase’ and ‘Consolidation phase’ represent the course of the control strategy for the counterfactual scenario.


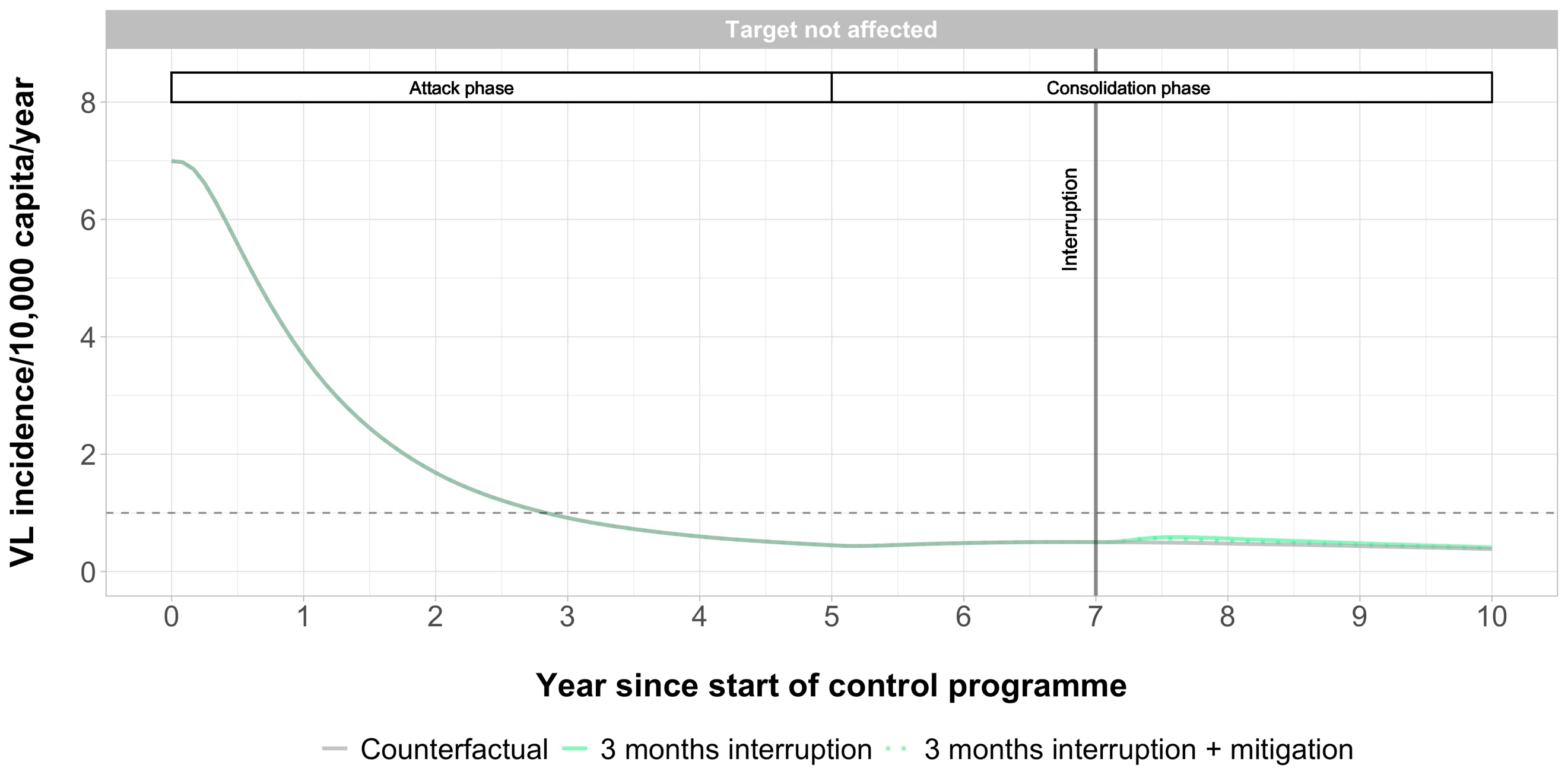


**Figure S5. Predicted visceral leishmaniasis incidence over time by Model E1.** An example of a typical Indian setting with a pre-control endemicity of 7 VL cases/10,000 capita/year with a 3-month interruption 7 years after the start of the control programme. The white bars at the top stating ‘Attack phase’ and ‘Consolidation phase’ represent the course of the control strategy for the counterfactual scenario.

**
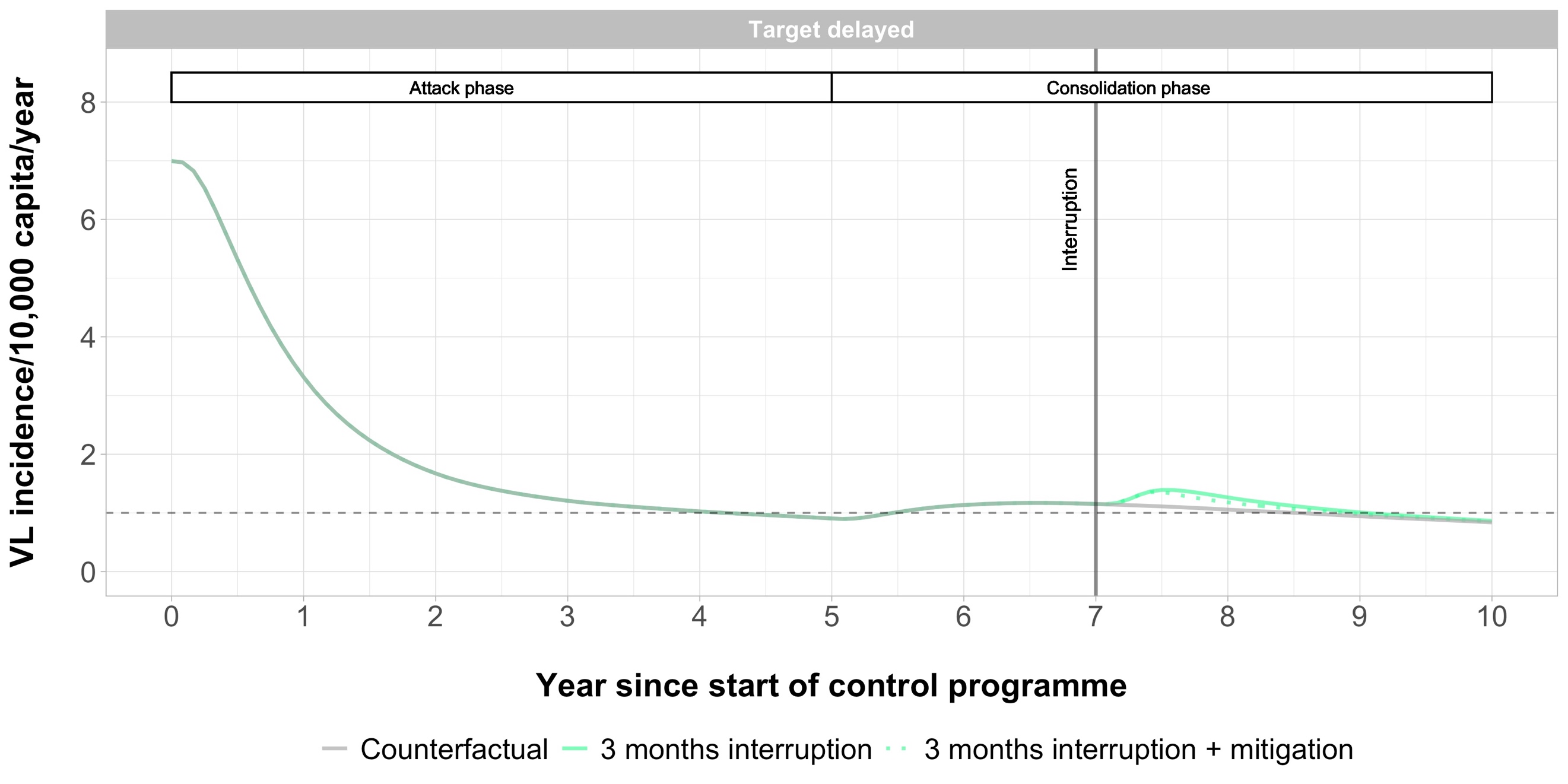
**

**Figure S6. Predicted visceral leishmaniasis incidence over time by Model E0.** An example of a typical Indian setting with a pre-control endemicity of 7 VL cases/10,000 capita/year with a 3-month interruption 7 years after the start of the control programme. The white bars at the top stating ‘Attack phase’ and ‘Consolidation phase’ represent the course of the control strategy for the counterfactual scenario.

## 5.3 Differences in time to achieving VL elimination for 3 alternative durations of interruption

The outliers that are present in Panel B of Figures SI-6, and SI-7 as well as in Figure 2 from the main text with are caused by the mitigation strategy leading to just not losing the elimination target whereas in the interruption scenario it does.

**Figure S7. Model E1: Heatmaps presenting the differences in time to achieving the VL elimination target for three combinations of scenarios (in years) with a 6-month interruption.** Panel A: interruption vs. counterfactual; Panel B: mitigation vs interruption, and Panel C: mitigation vs counterfactual. The letters D, L, and N correspond to the impact on the target when comparing the counterfactual scenario to the interruption scenario; delayed, lost, or not affected.

**Figure S8. Model E0: Heatmaps presenting the differences in time to achieving the VL elimination target for three combinations of scenarios (in years) with a 6-month interruption.** Panel A: interruption vs. counterfactual; Panel B: mitigation vs interruption, and Panel C: mitigation vs counterfactual. The letters D, L, and N correspond to the impact on the target when comparing the counterfactual scenario to the interruption scenario; delayed, lost, or not affected.

**Figure S9. Model E0: Heatmaps presenting the differences in time to achieving the VL elimination target for three combinations of scenarios (in years) with a 12-month interruption.** Panel A: interruption vs. counterfactual; Panel B: mitigation vs interruption, and Panel C: mitigation vs counterfactual. The letters D, L, and N correspond to the impact on the target when comparing the counterfactual scenario to the interruption scenario; delayed, lost, or not affected.

**Figure S10. Model E1: Heatmaps presenting the differences in time to achieving the VL elimination target for three combinations of scenarios (in years) with an 18-month interruption.** Panel A: interruption vs. counterfactual; Panel B: mitigation vs interruption, and Panel C: mitigation vs counterfactual. The letters D, L, and N correspond to the impact on the target when comparing the counterfactual scenario to the interruption scenario; delayed, lost, or not affected.

**Figure S11. Model E0: Heatmaps presenting the differences in time to achieving the VL elimination target for three combinations of scenarios (in years) with an 18-month interruption.** Panel A: interruption vs. counterfactual; Panel B: mitigation vs interruption, and Panel C: mitigation vs counterfactual. The letters D, L, and N correspond to the impact on the target when comparing the counterfactual scenario to the interruption scenario; delayed, lost, or not affected.

**Figure S12. Model E1: Heatmaps presenting the differences in time to achieving the VL elimination target for three combinations of scenarios (in years) with a 24-month interruption.** Panel A: interruption vs. counterfactual; Panel B: mitigation vs interruption, and Panel C: mitigation vs counterfactual. The letters D, L, and N correspond to the impact on the target when comparing the counterfactual scenario to the interruption scenario; delayed, lost, or not affected.

**Figure S13. Model E0: Heatmaps presenting the differences in time to achieving the VL elimination target for three combinations of scenarios (in years) with a 24-month interruption.** Panel A: interruption vs. counterfactual; Panel B: mitigation vs interruption, and Panel C: mitigation vs counterfactual. The letters D, L, and N correspond to the impact on the target when comparing the counterfactual scenario to the interruption scenario; delayed, lost, or not affected.

## 5.4 Differences in cumulative VL incidence for 3 alternative durations of interruption

**Figure S14. Model E1: Heatmaps presenting the differences in cumulative incidence (VL cases/10,000) to achieving the VL elimination target for three combinations of scenarios with a 6-month interruption.** Panel A: interruption vs. counterfactual; Panel B: mitigation vs interruption, and Panel C: mitigation vs counterfactual. We use a different colour in panel B to indicate the finer scale relative to that depicted in panels A and C. The letters D, L, and N correspond to the impact on the target when comparing the counterfactual scenario to the interruption scenario; delayed, lost, or not affected.

**Figure S15. Model E0: Heatmaps presenting the differences in cumulative incidence (VL cases/10,000) to achieving the VL elimination target for three combinations of scenarios with a 6-month interruption.** Panel A: interruption vs. counterfactual; Panel B: mitigation vs interruption, and Panel C: mitigation vs counterfactual. We use a different colour in panel B to indicate the finer scale relative to that depicted in panels A and C. The letters D, L, and N correspond to the impact on the target when comparing the counterfactual scenario to the interruption scenario; delayed, lost, or not affected.

**Figure S16. Model E0: Heatmaps presenting the differences in cumulative incidence (VL cases/10,000) to achieving the VL elimination target for three combinations of scenarios with a 12-month interruption.** Panel A: interruption vs. counterfactual; Panel B: mitigation vs interruption, and Panel C: mitigation vs counterfactual. We use a different colour in panel B to indicate the finer scale relative to that depicted in panels A and C. The letters D, L, and N correspond to the impact on the target when comparing the counterfactual scenario to the interruption scenario; delayed, lost, or not affected.

**Figure S17. Model E1: Heatmaps presenting the differences in cumulative incidence (VL cases/10,000) to achieving the VL elimination target for three combinations of scenarios with an 18-month interruption.** Panel A: interruption vs. counterfactual; Panel B: mitigation vs interruption, and Panel C: mitigation vs counterfactual. We use a different colour in panel B to indicate the finer scale relative to that depicted in panels A and C. The letters D, L, and N correspond to the impact on the target when comparing the counterfactual scenario to the interruption scenario; delayed, lost, or not affected.

**Figure S18. Model E0: Heatmaps presenting the differences in cumulative incidence (VL cases/10,000) to achieving the VL elimination target for three combinations of scenarios with an 18-month interruption.** Panel A: interruption vs. counterfactual; Panel B: mitigation vs interruption, and Panel C: mitigation vs counterfactual. We use a different colour in panel B to indicate the finer scale relative to that depicted in panels A and C. The letters D, L, and N correspond to the impact on the target when comparing the counterfactual scenario to the interruption scenario; delayed, lost, or not affected.

**Figure S19. Model E1: Heatmaps presenting the differences in cumulative incidence (VL cases/10,000) to achieving the VL elimination target for three combinations of scenarios with a 24-month interruption.** Panel A: interruption vs. counterfactual; Panel B: mitigation vs interruption, and Panel C: mitigation vs counterfactual. We use a different colour in panel B to indicate the finer scale relative to that depicted in panels A and C. The letters D, L, and N correspond to the impact on the target when comparing the counterfactual scenario to the interruption scenario; delayed, lost, or not affected.

**Figure S20. Model E0: Heatmaps presenting the differences in cumulative incidence (VL cases/10,000) to achieving the VL elimination target for three combinations of scenarios with a 24-month interruption.** Panel A: interruption vs. counterfactual; Panel B: mitigation vs interruption, and Panel C: mitigation vs counterfactual. We use a different colour in panel B to indicate the finer scale relative to that depicted in panels A and C. The letters D, L, and N correspond to the impact on the target when comparing the counterfactual scenario to the interruption scenario; delayed, lost, or not affected.

**Figure S21. Zoom in of VL incidence over time for a setting with a pre-control endemicity of 9/10,000/year with a 1-year interruption of the control programme 9 years after the start of the programme (Model E1).** The orange line represents the default scenario, the red line the interruption scenario, and the green line the mitigation scenario. The horizontal grey dashed line represents the 1/10,000/year elimination target. The pink vertical line represents the timing of the interruption.

# 5. References

1. World Bank. Crude birth rate per 1,000 population (India, 2011). http://data.worldbank.org/indicator. Accessed 4 Sep 2015. 2011.

2. India Ministry of Health and Family Welfare. Health and Family Welfare Statistics in India. https://data.gov.in/catalog/estimated-age-specific-death-rates-sex#web_catalog_tabs_block_10. Accessed 4 Sep 2015. 2013.

3. Le Rutte EA, Coffeng LE, Bontje DM, et al. Feasibility of eliminating visceral leishmaniasis from the Indian subcontinent: Explorations with a set of deterministic age-structured transmission models Quantitative analysis of strategies to achieve the 2020 goals for neglected tropical diseases: Wher. *Parasites and Vectors*. 2016;9(1). doi:10.1186/s13071-016-1292-0

4. Le Rutte EA, Chapman LAC, Coffeng LE, et al. Elimination of visceral leishmaniasis in the Indian subcontinent: a comparison of predictions from three transmission models. *Epidemics*. 2017;18:67-80. doi:10.1016/j.epidem.2017.01.002

5. Jervis S, Chapman LAC, Dwivedi S, et al. Variations in visceral leishmaniasis burden, mortality and the pathway to care within Bihar, India. *Parasit Vectors*. 2017;10(1):601. doi:10.1186/s13071-017-2530-9

6. Le Rutte EA, Chapman LAC, Coffeng LE, et al. Policy Recommendations From Transmission Modeling for the Elimination of Visceral Leishmaniasis in the Indian Subcontinent. *Clin Infect Dis*. 2018;66(suppl_4):S301-S308. doi:10.1093/cid/ciy007

7. Maintz E-M, Hassan M, Huda MM, et al. Introducing single dose liposomal amphotericin B for the treatment of visceral leishmaniasis in rural bangladesh: feasibility and acceptance to patients and health staff. *J Trop Med*. 2014;2014:676817. doi:10.1155/2014/676817

8. Sundar S, Sinha PK, Rai M, et al. Comparison of short-course multidrug treatment with standard therapy for visceral leishmaniasis in India: An open-label, non-inferiority, randomised controlled trial. *Lancet*. 2011;377(9764):477-486. doi:10.1016/S0140-6736(10)62050-8

9. Rahman KM, Islam S, Rahman MW, et al. Increasing Incidence of Post–Kala‐Azar Dermal Leishmaniasis in a Population‐Based Study in Bangladesh. *Clin Infect Dis*. 2010;50(1):73-76. doi:10.1086/648727

10. Ramesh V, Kaushal H, Mishra AK, Singh R, Salotra P. Clinico-epidemiological analysis of Post kala-azar dermal leishmaniasis (PKDL) cases in India over last two decades: a hospital based retrospective study. *BMC Public Health*. 2015;15(1):1092. doi:10.1186/s12889-015-2424-8

11. Uranw S, Ostyn B, Rijal A, et al. Post-Kala-azar Dermal Leishmaniasis in Nepal: A Retrospective Cohort Study (2000-2010). *PLoS Negl Trop Dis*. 2011;5(12).

12. Mondal D, Bern C, Ghosh D, et al. Quantifying the Infectiousness of Post-Kala-Azar Dermal Leishmaniasis Toward Sand Flies. *Clin Infect Dis*. 2019;69(2):251-258. doi:10.1093/cid/ciy891

13. Le Rutte EA, Zijlstra EE, de Vlas SJ. Post-Kala-Azar Dermal Leishmaniasis as a Reservoir for Visceral Leishmaniasis Transmission. *Trends Parasitol*. 2019;35(8):590-592. doi:10.1016/j.pt.2019.06.007

14. Stauch A, Sarkar RR, Picado A, et al. Visceral leishmaniasis in the Indian subcontinent: modelling epidemiology and control. *PLoS Negl Trop Dis*. 2011;5(11):e1405. doi:10.1371/journal.pntd.0001405

15. Singh RP, Picado A, Alam S, et al. Post-kala-azar dermal leishmaniasis in visceral leishmaniasis-endemic communities in Bihar, India. *Trop Med Int Heal*. 2012;17(11):1345-1348. doi:10.1111/j.1365-3156.2012.03067.x

16. Desjeux P, Ghosh RS, Dhalaria P, Strub-Wourgaft N, Zijlstra EE. Report of the Post Kala-azar Dermal Leishmaniasis (PKDL) Consortium Meeting, New Delhi, India, 27-29 June 2012. *Parasit Vectors*. 2013;6(1):196. doi:10.1186/1756-3305-6-196

17. Kirk R LD. Studies in leishmaniasis in the Anglo-Egyptian Sudan. Phlebotomus in relation to leishmaniasis in the Sudan. *Trans R Soc Trop Med Hyg*. 1955;49:229-240.

18. Palit A, Bhattacharya SK, Kundu S. Gonotrophic cycle and age gradation of Phlebotomus argentipes in West Bengal , India. 2011.

19. Sacks DL, Perkins PV. Development of infective stage Leishmania promastigotes within phlebotomine sand flies. *Am J Trop Med Hyg*. 1985;34:456–459.

20. Hati A, Sur S, De N, et al. Longitudinal study on distribution of Phlebotomus argentipes sandflies at different heights in cattleshed. *Indian J Med Res*. 1991;93:388–390.

21. Hati. AK, Palit A, Chakraborty S, S. Bhattacharya KKG and, S.Das. PHLEBOTOMUS ARGENTIPES ANNANDALE AND BRUNETTI (DIPTERA) CAUGHT ON MAN AT NIGHT IN A CLEAN BIOTOPE. *Rec, zool, Surv India*. 1984;81:9-12.

22. Behrend MR, Basáñez M-G, Hamley JID, et al. Modelling for policy: The five principles of the Neglected Tropical Diseases Modelling Consortium. Blanton J, ed. *PLoS Negl Trop Dis*. 2020;14(4):e0008033. doi:10.1371/journal.pntd.0008033
